# Are omicron infections more frequently associated with bacterial co-infections?

**DOI:** 10.5578/tt.202402885

**Published:** 2024-06-12

**Authors:** Faig TEYMURLU, Yaşar Selim SARSILMAZ, Özge GÜZEL AYGÖREN, Beltinge GÜLTEKİN, Şeymanur GÜL, Aslı SUNER, Abdullah SAYINER

**Affiliations:** 1 Department of Chest Diseases, Ege University Faculty of Medicine, İzmir, Türkiye; 2 Department of Biostatistics and Medical Informatics, Ege University Faculty of Medicine, İzmir, Türkiye

## Abstract

**ABSTRACT**

**
Are omicron infections more frequently associated with bacterial co-infections?
**

**Introduction:** *Clinical observations suggest that Omicron infections may pre- sent with different radiographic findings and be more frequently associated with bacterial co-infections, but there is a paucity of published data. This study aimed to compare the clinical and radiographic findings of patients hospitalized with Omicron versus alpha-delta infections.
*

**Materials and Methods:** *Between January 1, 2021 and June 30, 2021 (alpha
*

*
and delta period) and between January 1, 2022 and July 31, 2022 (Omicron period), respectively 149 and 163 COVID-19 PCR-positive patients who were followed up in the COVID-19 ward and intensive care unit of a tertiary care center were included in the study. Clinical (presence of fever and purulent sputum), laboratory and radiologic findings of the two groups were com- pared. Sputum culture results and antibiotic use were also evaluated.
*

**Results:** *In the alpha/delta group, ground glass opacities were seen in 75.2%
*

*
(112) of the patients, consolidation in 2.7% (4), and both findings together in 6.0% (9). In the Omicron group, ground glass was seen in 40.5% (66), con- solidation in 5.5% (9), and both ground glass and consolidation together in 8.7% (13) (p< 0.001). Procalcitonin levels were 0.25 µg/L or higher in 29.6% and 43.9% of the patients in the alpha/delta and Omicron groups, respec- tively. Mean PCT values were 0.36 µg/L and 1.93 µg/L, respectively (p> 0.05). CRP levels were similar in both groups. Mean LDH level in the Omicron group was 278 U/L and was significantly lower than the alpha/delta group (381 U/L) (p< 0.001). The proportion of patients requiring intensive care during hospitalization was higher in the alpha/delta group (36.2% vs 26.4%) (p= 0.06).
*

**Conclusion:** *Lower LDH levels, less need for intensive care and less frequent development of ARDS indicate that Omicron causes milder disease, while a higher rate of consolidation and higher procalcitonin levels suggest a higher frequency of bacterial co-infections.
*

**Key words:** *COVID-19; SARS-CoV-2; co-infections
*

**ÖZ**

**
Omicron enfeksiyonlarında bakteriyel koenfeksiyon riski daha yüksek mi?
**

**Giriş:** *Klinik gözlemler, Omicron enfeksiyonlarının farklı radyografik bulgularla ortaya çıkabileceğini ve bakteriyel koenfeksiyonlarla daha sık ilişkili olabileceğini düşündürmektedir, ancak yayımlanmış veri azdır. Bu çalışmanın amacı, Omicron ve alfa-delta enfeksiyon- ları ile hastaneye yatırılan hastaların klinik ve radyografik bulgularını karşılaştırmaktır.
*

**Materyal ve Metod:** *1 Ocak 2021-30 Haziran 2021 tarihleri arasında (alfa ve delta dönemi) ve 1 Ocak 2022-31 Temmuz 2022 tarihleri arasında (Omicron dönemi) üçüncü basamak bir merkezin COVID-19 servisinde ve yoğun bakım ünitesinde takip edilen 149 ve 163 COVID-19 PCR-pozitif hasta çalışmaya dahil edilmiştir. İki grubun klinik (ateş ve pürülan balgam varlığı), laboratuvar ve rad- yolojik bulguları karşılaştırılmıştır. Balgam kültürü sonuçları ve antibiyotik kullanımı da değerlendirilmiştir.
*

**Bulgular:** *Alfa/delta grubunda hastaların %75,2 (112)’sinde buzlu cam opasiteleri, %2,7 (4)’sinde konsolidasyon ve %6,0 (9)’sında her iki bulgu birlikte görülmüştür. Omicron grubunda ise %40,5 (66)’inde buzlu cam, %5,5 (9)’inde konsolidasyon ve %8,7 (13)’sinde hem buzlu cam hem de konsolidasyon birlikte görüldü (p< 0,001). Prokalsitonin düzeyleri alfa/delta ve Omicron grupla- rında hastaların %29,6 ve %43,9’unda 0,25 µg/L veya üzerindeydi. İki gruptaki ortalama PCT değerleri sırasıyla 0,36 µg/L ve 1,93 µg/L idi (p> 0.05). CRP değerleri iki grupta da benzer düzeydeydi. Ortalama LDH düzeyi Omicron grubunda 278 U/L idi ve alfa/ delta grubuna (381 U/L) göre daha düşüktü (p< 0,001). Yoğun bakım gerektiren hasta oranı alfa/delta grubunda daha yüksekti (%36,2 vs 26,4) (p= 0,06).
*

**Sonuç:** *Daha düşük LDH seviyeleri, daha az yoğun bakım ünitesi ihtiyacı ve daha az ARDS gelişimi Omicron’un daha hafif hastalığa neden olduğunu gösterirken, daha yüksek konsolidasyon oranı ve daha yüksek prokalsitonin seviyeleri daha yüksek bakteriyel koen- feksiyon sıklığına işaret etmektedir.
*

**Anahtar kelimeler:** *COVID-19; SARS-CoV-2; bakteriyel koenfeksiyon
*

## INTRODUCTION

As of today, SARS-CoV-2 infections have led to more than 17 million documented cases and more than

101.492 deaths in Türkiye according to the official sources (1,2).

Different variants originating from mutations along the course of the disease have been observed. The most prevalent and concerning ones have been the alpha, delta and Omicron variants. The World Health Organization announced on November 24, 2021 that a new variant (B.1.1.529) was active and labeled it as Omicron. When compared with the previous vari- ants, Omicron was observed to be more infectious (3). Omicron variant has been diagnosed in Türkiye starting from December 2021. There was a rapid increase in the number of infected cases, which was associated with a relatively weak increase in hospi- talizations and mortality (2,4). Chronologically, the alpha variant became the dominant strain in November 2020 and its activity lasted till April 2021. Then, delta activity started and became the dominant strain in a short period. Since December 2021, Omicron activity has been mainly observed Eris, or EG.5.1, the current dominant strain, is considered to be an Omicron subvariant (5).

In a meta-analysis of studies performed during the period when alpha and delta variants were dominant (2020 and 2021), the most frequently observed CT finding was bilateral, subpleural, multifocal ground glass opacities (6). Besides, the frequency of bacterial

co-infections associated with COVID-19 during the same period was reported to be 5-11% (7,8). In con- trast, we observed in patients who presented to our hospital during the Omicron period that the radio- graphic findings were somewhat different and antibi- otics were more frequently used in relation with the laboratory and radiographic features.

There are scant data on the clinical and radiographic findings of Omicron infections. The aim of this study was to define the radiographic features of and the frequency of bacterial co-infections in Omicron infections in comparison with the alpha and delta variant infections.

## MATERIALS and METHODS

**Study Population**

Between January 1, 2021 and June 30, 2021 (alpha and delta period) and between January 1, 2022 and July 31, 2022 (Omicron period), respectively 149 and 163 COVID-19 PCR-positive patients who were fol- lowed up in the COVID-19 ward and intensive care unit of the Department of Chest Diseases, Ege University Faculty of Medicine were included in the study.

The study protocol conforms to the ethical guidelines of the 1964 Declaration of Helsinki and its later amendments, and it was approved by Institutional Ethics Committee of Ege University Faculty of Medicine (20-5T/48). Written informed consent was obtained from all patients during the time they were

hospitalized for their clinical data to be registered in the database and used anonymously for scientific purposes.

## Parameters Assessed for the Comparison of the Two Periods

Clinical (presence of fever and purulent sputum), laboratory (serum CRP, procalcitonin, LDH levels, leukocyte and neutrophil counts) and radiologic find- ings (presence of ground glass opacities and consoli- dation) of the two groups were compared. High fever was defined as a temperature of 37.5 °C or higher as determined by an ear thermometer. Sputum culture results and antibiotic use were also evaluated.

## Statistical Analysis

Descriptive statistics were used for demographic data. The conformity of the variables to normal distri- bution was examined using visual (histogram and probability graphs) and analytical methods (Kolmogorov-Smirnov / Shapiro-Wilk tests). For non- normally distributed variables, median and interquar- tile range were used (using frequency tables for ordinal variables). For normally distributed variables, mean and standard deviation were used. Chi-square and Mann-Whitney U tests were used to compare independent groups for non-normally distributed data and ordinal and nominal data, and Student’s t test was used to compare normally distributed data. Univariate logistic regression and multivariate logis- tic regression analysis were used to determine inde- pendent variables associated with mortality. Results were considered significant when the p value was below 0.05.

## RESULTS

In the alpha-delta and Omicron periods, 51.7% (77) and 56.4% (92) of the patients were males, respec- tively (p> 0.05). The demographic features and comorbidities of the two study groups are shown in Table.

At the time of hospitalization, 66.4% (99) of the patients were hospitalized in the ward and 33.6%

(50) in the intensive care unit in 2021, whereas in 2022, 82.8% (135) were hospitalized in the ward and 17.2% (28) in the intensive care unit (p< 0.001).

## Clinical Findings at Admission

Upon admission, 24.8% (37) of the patients in the alpha/delta group and 9.8% (16) of the patients in the Omicron group had high fever (p< 0.001).

There was no difference in the oxygenation status of the patients at admission; 73.2% (109) in the alpha- delta group and 71.2% (116) in the Omicron group needed oxygen.

## CT Findings

In the alpha/delta group, ground glass opacities were seen in 75.2% (112) of the patients, consolidation in 2.7% (4), and both findings together in 6.0% (9). In the Omicron group, ground glass was seen in 40.5% (66), consolidation in 5.5% (9), and both ground glass and consolidation together in 8.7% (13) (p< 0.001). No radiographic lung involvement was observed in 6.7% (10) of the patients in the alpha/ delta group and 1.8% (3) in the Omicron group.

**Table:** 

**Table 1.** Comparison of demographic and clinical characteristics		
	**Alpha/delta**	**Omicron**
Age	61.15 ± 15.05	71.51 ± 13.88
Male	51.7% (77)	56.4% (92)
Presence of comorbidities	77.18% (115)	90.80% (148)
Hypertension	56.52% (65)	57.43% (85)
Coronary artery disease*	13.04% (15)	24.32% (36)
Congestive heart failure	24.35% (28)	15.54% (23)
Diabetes mellitus**	31.30% (36)	37.84% (56)
COPD***	7.83% (9)	23.65% (35)
Malignancy	22.61% (26)	20.27% (30)
*p< 0.05. **p< 0.05. ***p< 0.001		

**Table:** 

**Table 2.** Comparison of markers of infection and inflammation						
** Markers of infection and inflammation **						
	**Procalcitonin (µg/L) (p> 0.05)**		**CRP (mg/L) (p> 0.05)**		LDH (U/L)*	
	** Percentage of patients with levels >0.25 µg/L **	**Mean ± SD**	** Percentage of patients with levels >5 mg/L **	**Mean ± SD**	** Percentage of patients with levels >225 U/L **	**Mean ± SD**
Alpha-delta period	29.6%	0.36 ± 0.70	93.2%	96.84 ± 87.63	%88.2	381 ± 165
Omicron period	43.9%	1.93 ± 9.11	95.0%	84.15 ± 75.38	%68.0	278 ± 97
*p< 0.001.						

## Biochemical Parameters

Procalcitonin (PCT) was measured in 54.4% of the patients in the alpha/delta group and 65.6% of the patients in the Omicron group. Procalcitonin values were 0.25 µg/L or higher in 29.6% and 43.9% of the patients, respectively. Mean PCT values in the two groups were 0.36 µg/L and 1.93 µg/L, respectively (p> 0.05) (Table 2).

C-Reactive Protein (CRP) values were measured in almost all patients (99.3% and 98.2%). Mean CRP value was 96.8 mg/L in the alpha/delta group and

84.2 mg/L in the Omicron group (p> 0.05). The pro- portions of patients with CRP values above 5.0 mg/L were 93.2% and 95.0%.

Mean leukocyte counts were 8.82 10^3^/µL in the alpha/delta group and 9.28 10^3^/µL in the Omicron group while mean neutrophil counts were 6.91 10^3^/ µL and 6.88 10^3^/µL, respectively (p> 0.05).

Lactate dehydrogenase (LDH) levels were examined in 63.8% of the patients in the alpha/delta group and were found to be 225 U/L and above in 88.2%. Mean LDH level was 381 U/L. In the Omicron group, LDH was checked in 28.8% of the patients and values of 225 U/L and above were detected in 68.0%. Mean LDH value was 278 U/L and was significantly lower than the alpha/delta group (p< 0.001).

Of the 45 patients with PCT >0.25 in the Omicron period, 77.78% (35) received antibiotherapy, 31.11%

(14) needed ICU during follow-up and 15.56% (7) died. Mean length of hospitalization was 11.0 days. In the group who did not receive antibiotherapy, 20%

(10) patients needed ICU during follow-up and 20%

(2) of these patients died. Mean length of hospitaliza- tion was 5.3 days.

When these two groups were compared in terms of ICU requirement (p= 0.39) and mortality (p= 0.66)

rates, no statistically significant difference was observed.

## Sputum Cultures

At the time of hospital admission, 32 (21.5%) and 43 (26.4%) patients had sputum complaints in the alpha-delta and Omicron groups, respectively. Sputum samples could only be obtained from a small proportion of the patients (23.5% in alpha/delta period - 2021 and 12.3% in Omicron period - 2022). Sputum culture was positive in 9.4% (14) of the patients in 2021, and 8.6% (14) of the patients in 2022 (p> 0.05).

## Antibiotic Treatment

Antibiotic treatment was given to 45.6% (68) patients in the alpha/delta group and 67.5% (110) patients in the Omicron group (p< 0.001).

## Clinical Course

The proportion of patients requiring intensive care during the entire hospitalization was 36.2% (54) and 26.4% (43) in the two groups, respectively (p= 0.06). The proportions of patients who needed invasive mechanical ventilation in the intensive care unit were 11.4% (17) and 9.2% (15) (p= 0.52), and the proportions of patients who needed non-invasive mechanical ventilation were 21.5% (32) and 19.6% (32), respectively (p= 0.68). Mean duration of inva- sive mechanical ventilation was 7.9 and 4.6 days, respectively (p= 0.022).

In the alpha/delta group, 85.2% (127) and in the Omicron group, 87.7% (143) of the patients were discharged after completion of treatment. Of the patients, 14.8% (22) and 12.3% (20) died due to dif- ferent causes during treatment, respectively (p> 0.05).

Mean length of hospital stay was similar in both groups (8.8 and 9.3 days, respectively).

## DISCUSSION

The most important findings of this study are the different clinical and radiographic findings of COVID- 19 infections during the Omicron period compared to previous periods. In the Omicron period, peripheral opacities with ground-glass pattern were observed more rarely and consolidations were observed more frequently. In parallel, mean procalcitonin level was relatively higher in Omicron infections, but the difference between the two groups did not reach statistical significance. Because of these findings, attending physicians more frequently considered bacterial co-infection and administered antibiotic treatment during the Omicron period.

There is no bacteriologic evidence that bacteria caused more frequent co-infections in the Omicron period. The lack of a higher rate of growth in bacte- riologic cultures is due to the fact that sputum cul- tures could only be performed in a very small propor- tion of patients. In addition, in some of the cultures that could be performed, respiratory specimens were obtained after antibiotic treatment was started, and this probably contributed to low growth rates. Low culture positivity rates are in accordance with a pre- vious large study, which showed that sputum and/or blood cultures were positive in 12.6% of CAP patients only (9).

Higher LDH and ferritin levels and relatively higher CRP levels in the alpha/delta group, although not statistically significant, may indicate a more severe inflammatory process. In relation to these data, the rate of intensive care unit admission was higher in alpha/delta infections at the time of hospitalization. When the entire hospital stay was considered, the proportion of patients requiring intensive care during the Omicron period was still lower, although this was above the significance threshold. This latter observa- tion possibly indicates that Omicron patients with more comorbidities had an unfavorable clinical course during their hospital follow-up.

One of the limitations of this study was that the causes of mortality were not recorded. Similar mor- tality rates in the two periods were possibly influ- enced by the differences in the severity of pulmonary involvement (worse in alpha/delta period) versus the differences in patient profiles, namely underlying medical conditions and frailty (worse in the Omicron period). As is known, the prognosis of most of the

patients hospitalized during the Omicron period was mainly determined by their age and underlying chronic diseases (10,11). Another important limita- tion was that we were not able to get the data of all COVID-19 patients who presented to the emergency department or the outpatient clinic during the same time intervals. We have thus not been able to esti- mate the proportions of patients with mild versus moderate-to-severe disease, which would have given a better idea about the severity of the infections due to the three variants.

In conclusion, Omicron infections are characterized by different radiographic and laboratory features than infections due to previous variants and require more frequent antibiotic treatment.

**Ethical Committee Approval:** This study was approved by the Ege University Faculty of Medicine Clinical Research Committee (Decision no: 20-5T/48, Date: 15.05.2020).

## CONFLICT of INTEREST

The authors declare that they have no conflict of interest.

## AUTHORSHIP CONTRIBUTIONS

Concept/Design: AS Analys/Interpretation: FT, ASK

Data acqusition: BG, FT, YSS, ÖGA, ŞG Writing: FT, AS

Clinical Revision: AS, FT, ÖGA, YSS, BG, ŞG Final Approval: AS, FT, ÖGA, YSS, BG, ŞG

## REFERENCES

1 *
  World Health Organization (WHO). WHO coronavirus (COVID‐19) dashboard. Accessed date: 2022 Nov 29. Available from: https://covid19.who.int/181
  *
2 *
  T.C. Sağlık Bakanlığı COVID-19 bilgilendirme platformu. Available from: https://covid19.saglik.gov.tr/
  *
3 *
  World Health Organization (WHO). Classification of Omicron (B.1.1.529): SARS‐CoV‐2 variant of concern. Accessed date: 2022 Nov 29. Available from: https:// www.who.int/news/item/26-11-2021-classification-of-omicron-(b.1.1.529)-sars-cov-2-variant-of-concern*
4 *
  Türkiye Sağlık Bakanlığı. Türkiye’de baskın hale gelen omi- cron varyantının son 10 günlük seyrine ilişkin açıklama Accessed date: 31.12.2021. Available from: http://www/saglik.gov.tr/TR,87028/turkiyede-baskin-hale-gelen-Omi-cron-varyantinin-son-10-gunluk-seyrine-iliskin-aciklama-31122021.html.
  *
5 *
  Worldometer. New coronavirus cases in Turkey. Available from: http://www.worldometers.info/coronavirus/coun-virus/country/turkey*
6 *
  Zhou X, Pu Y, Zhang D, Xia Y, Guan Y, Liu S, et al. CT find- ings and dynamic imaging changes of COVID-19 in 2908 patients: A systematic review and meta-analysis. Acta Radiol 2022; 63(3): 291-310. https://doi.org/10.1177/0284185121992655*
7 *
  Calderon M, Gysin G, Gujjar A, McMaster A, King L, Comandé D, et al. Bacterial co-infection and antibiotic stewardship in patients with COVID-19: A systematic review and meta-analysis. BMC Infect Dis 2023; 23(1): 14. https://doi.org/10.1186/s12879-022-07942-x*
8 *
  Alshaikh FS, Godman B, Sindi ON, Seaton RA, Kurdi A. Prevalence of bacterial co-infection and patterns of antibi- otics prescribing in patients with COVID-19: A systematic review and meta-analysis. PLoS One. 2022; 17(8): e0272375. https://doi.org/10.1371/journal.pone.0272375*
9 *
  Çilli A, Sayıner A, Çelenk B, Şakar Coşkun A, Kılınç O, Hazar A, et al. Antibiotic treatment outcomes in commu- nity-acquired pneumonia. Turk J Med Sci 2018; 48: 730-6. https://doi.org/10.3906/sag-1709-144*
10 *
  Candelli M, Sacco Fernandez M, Pignataro G, Merra G, Tullo G, Bronzino A, et al. ANCOC score to predict mor- tality in different SARS-CoV-2 variants and vaccination status. J Clin Med 2023; 12(18): 5838. https://doi.org/10.3390/jcm12185838*
11 *
  Giannitsioti E, Mavroudis P, Speggos I, Katsoulidou A, Pantazis N, Loupis T, et al. Real life treatment experience and outcome of consecutively hospitalised patients with SARS-CoV-2 pneumonia by Omicron-1 vs Delta variants. Infect Dis (Lond) 2023; 55(10): 706-15. https://doi.org/10.1080/23744235.2023.2232445*

